# Supplementary material for: ProteinShader: illustrative rendering of macromolecules
Source: BMC Struct Biol. 2009 Mar 30;9:19. doi: 10.1186/1472-6807-9-19 (PMC2672931; doi:10.1186/1472-6807-9-19)
Supplement: Additional file 1 — ProteinShader program without source code. This compressed file contains the complete ProteinShader program including associated libraries, but no source code. A README.txt file gives an overview of the ProteinShader distribution, and the index.html file in the help subdirectory has directions on getting started with the program as well as a set of tutorials. [file 1472-6807-9-19-S1.zip › ProteinShader-beta-0_9_4-binary/help/api/org/proteinshader/graphics/shader/ShaderProgramFactory.html]

ShaderProgramFactory (ProteinShader API)


|  |  |  |
| --- | --- | --- |
| **Overview** \| **Package** \| **Class** \| **Use** \| **Tree** \| **Deprecated** \| **Index** \| **Help** | |  |
| **PREV CLASS**   NEXT CLASS | **FRAMES**    **NO FRAMES**     **All Classes** |  |
| SUMMARY: NESTED \| FIELD \| CONSTR \| METHOD | DETAIL: FIELD \| CONSTR \| METHOD |  |


---


## org.proteinshader.graphics.shader Class ShaderProgramFactory

```
java.lang.Object
  org.proteinshader.graphics.shader.ShaderProgramFactory
```

---

``` public class ShaderProgramFactory extends Object ```

Knows how to compile and link an OpenGL Shading Language vertex shader
and fragment shader to get an OpenGL "program" object.

---

| **Field Summary** | |
| --- | --- |
| `static String` | `SHADER_DIRECTORY`             Specifies the directory the OpenGL Shading Language vertex and fragment shaders are kept in. |


**Constructor Summary**

`ShaderProgramFactory()`
  
          Constructs a ShaderProgramFactory.


| **Method Summary** | |
| --- | --- |
| `ShaderProgram` | `compile(GL gl, ShaderEnum pair)`             Reads a pair of vertex and fragment shader files and then compiles and links them. |
| `List<ShaderProgram>` | `compileAllShaders(GL gl)`             Compiles all shaders pairs defined by the ShaderEnum. |
| `ShaderProgram` | `createShaderProgram(GL gl, File vertFile, File fragFile, ShaderEnum pair)`             Reads source code files for a vertex shader and a fragment shader and creates an OpenGL shader program object. |
| `ShaderProgram` | `createShaderProgram(GL gl, String vertFilename, String fragFilename, ShaderEnum pair)`             Reads source code files for a vertex shader and a fragment shader and creates an OpenGL shader program object. |
| `void` | `deleteShaderProgram(GL gl, ShaderProgram program)`             Calls delete on each OpenGL shader and program object name held by the ShaderProgram given as an argument. |
| `List<ShaderException>` | `getErrors()`             Returns a list with all ShaderExceptions that occurred during the last call to compileAllShaders(). |

**Methods inherited from class java.lang.Object**

`clone, equals, finalize, getClass, hashCode, notify, notifyAll, toString, wait, wait, wait`

**Field Detail**

### SHADER\_DIRECTORY

```
public static final String SHADER_DIRECTORY
```

:   Specifies the directory the OpenGL Shading Language
    vertex and fragment shaders are kept in.

    **See Also:**: Constant Field Values


**Constructor Detail**

### ShaderProgramFactory

```
public ShaderProgramFactory()
```

:   Constructs a ShaderProgramFactory.


**Method Detail**

### compileAllShaders

```
public List<ShaderProgram> compileAllShaders(GL gl)
```

:   Compiles all shaders pairs defined by the ShaderEnum. If the files for
    a pair cannot be found, an exception will be added to a list of exceptions
    that can be obtained by calling getErrors() right after using this method.

    :   **Parameters:**: `gl` - the current GL object. **Returns:**: A list with all of the ShaderProgram objects that could be created.

---


### getErrors

```
public List<ShaderException> getErrors()
```

:   Returns a list with all ShaderExceptions that occurred during the last call
    to compileAllShaders().

    :   **Returns:**: The list of errors.

---


### compile

```
public ShaderProgram compile(GL gl,
                             ShaderEnum pair)
                      throws ShaderException
```

:   Reads a pair of vertex and fragment shader files and then compiles
    and links them.

    :   **Parameters:**: `gl` - the current GL object.: `pair` - a ShaderEnum with the names of the vertex and fragment files. **Returns:**: A ShaderProgram object with information on an OpenGL Shading Language 'program' object. **Throws:**: `ShaderException` - if the shader program object cannot be obtained.

---


### createShaderProgram

```
public ShaderProgram createShaderProgram(GL gl,
                                         String vertFilename,
                                         String fragFilename,
                                         ShaderEnum pair)
                                  throws ShaderException
```

:   Reads source code files for a vertex shader and a fragment shader
    and creates an OpenGL shader program object.

    :   **Parameters:**: `gl` - the current GL object.: `vertFilename` - the file with the vertex shader code.: `fragFilename` - the file with the fragment shader code.: `pair` - a ShaderEnum with the names of the vertex and fragment files. **Returns:**: A ShaderProgram object that holds OpenGL references. **Throws:**: `ShaderException` - if the shader program object cannot be obtained.

---


### createShaderProgram

```
public ShaderProgram createShaderProgram(GL gl,
                                         File vertFile,
                                         File fragFile,
                                         ShaderEnum pair)
                                  throws ShaderException
```

:   Reads source code files for a vertex shader and a fragment shader
    and creates an OpenGL shader program object.

    :   **Parameters:**: `gl` - the current GL object.: `vertFile` - the file with the vertex shader code.: `fragFile` - the file with the fragment shader code.: `pair` - a ShaderEnum with the names of the vertex and fragment files. **Returns:**: A ShaderProgram object that holds OpenGL references. **Throws:**: `ShaderException` - if the shader program object cannot be obtained.

---


### deleteShaderProgram

```
public void deleteShaderProgram(GL gl,
                                ShaderProgram program)
```

:   Calls delete on each OpenGL shader and program object name held by
    the ShaderProgram given as an argument. If program is null or if
    a name happens to be zero, it will be silently ignored.

    :   **Parameters:**: `gl` - the current GL object.: `program` - the Java ShaderProgram object holding the OpenGL references.


---


|  |  |  |
| --- | --- | --- |
| **Overview** \| **Package** \| **Class** \| **Use** \| **Tree** \| **Deprecated** \| **Index** \| **Help** | |  |
| **PREV CLASS**   NEXT CLASS | **FRAMES**    **NO FRAMES**     **All Classes** |  |
| SUMMARY: NESTED \| FIELD \| CONSTR \| METHOD | DETAIL: FIELD \| CONSTR \| METHOD |  |


---

# *Copyright © 2007-2008*
